# Impact of patient-accessible electronic medical records in rheumatology: use, satisfaction and effects on empowerment among patients

**DOI:** 10.1186/1471-2474-15-102

**Published:** 2014-03-26

**Authors:** Rosalie van der Vaart, Constance HC Drossaert, Erik Taal, K Wiepke Drossaers-Bakker, Harald E Vonkeman, Mart AFJ van de Laar

**Affiliations:** 1 Department of Psychology, Health & Technology, University of Twente, Citadel H423, P.O. Box 217 7500 AE, Enschede, The Netherlands; 2 Arthritis Centre Twente, Medisch Spectrum Twente, Enschede, the Netherlands

**Keywords:** Electronic Medical Record, Web portal, Patient access, Patient empowerment, eHealth

## Abstract

**Background:**

To measure the use, satisfaction and impact of a web portal which provides patients with rheumatoid arthritis home access to their electronic medical records (EMR).

**Methods:**

A pretest-posttest study was conducted among 360 patients. Questionnaires assessed socio-demographics, health literacy, Internet use, disease characteristics, patient-provider relationship and empowerment before and after launching a hospital-based patient web portal. To measure the impact of the portal, patients’ satisfaction with care, trust in their rheumatologist, self-efficacy in patient-provider communication, illness perceptions, and medication adherence were assessed. The post-test included questions on portal use, satisfaction, and self-perceived impact due to portal use.

**Results:**

54% of respondents with Internet access had viewed their EMR. Respondents were positive about the ease of use and usefulness of the portal and reported very few problems. Age (*P* = .03), amount of Internet use (*P* = .01) and self-perceived Internet skills (*P* = .03) significantly predicted portal use. Of the respondents who had logged in, 44% reported feeling more involved in their treatment and 37% felt they had more knowledge about their treatment. Significant differences over time were not found on the empowerment-related instruments.

**Conclusions:**

The current portal succeeded in offering patients access to their EMR in a usable and understandable way. While its true impact is difficult to grasp, a relevant portion of the patients felt more involved in their treatment due to the web portal. Offering patients home EMR access, therefore, appears to be a valuable addition to the care process.

## Background

Since many rheumatic diseases are chronic and can have a large impact on patients’ lives, it is essential that patients become involved in their treatment and have proper self-care practices [1,2]. Increasing patients’ responsibilities and autonomy is also essential for the redesign of health services, from current disease- or institutional-centered models to patient-centered models of care, in order to keep expenses under control [3,4]. The implementation of information and communication technologies in health care can play an essential role in this shift [5]. A slowly emerging technology in health care is the ability to provide patients online home-access to their electronic medical records (EMRs), via hospital-based patient web portals [6,7]. The key benefit of this application is that patients can (repeatedly) read the documentation on their disease and treatment, at home, which has the potential to empower patients in their care process [8,9].

Providing patients home EMR-access may influence health care on several levels. First of all, it increases transparency of medical data, which could reduce medical errors, increase patients’ trust in care providers and could enhance patient satisfaction [10-12]. Secondly, patients’ knowledge and understanding of the disease and treatment may be enhanced [13], increasing their involvement in decision making processes [13,14]. Thirdly, patients may feel more control over their disease and treatment, which could positively influence treatment adherence [15], and even clinical outcomes [16].

While patient access to medical records could benefit health care, an online application might not suit everyone. Previous studies have shown that users of online applications are often relatively young and highly educated [17]. Furthermore, patients’ abilities to use (online) health information, also called “health literacy” is assumed to be related to acceptance of online applications [18]. Until now, little is known about the predictors for using home access to medical records or on the difficulties that patients experience when using this service.

**Figure 1:**
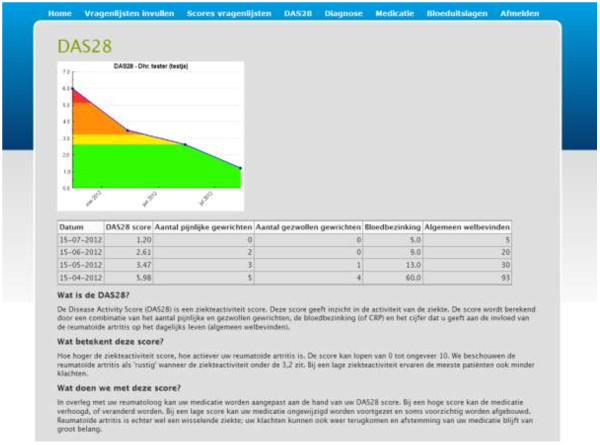
Screenshot of a login page providing rheumatoid arthritis disease activity (DAS28) scores with an explanation of the data.

Hospitals increasingly offer patients home access to their EMR, but to the best of our knowledge, no studies have thus far been conducted in the field of rheumatology. Still, previous studies have shown that patients are indeed interested in this option [19,20], and both rheumatologists and nurses believe that it could have a positive impact on the empowerment of their patients [21]. Based upon these studies, a web portal was designed following user-centered design principles [22], which offers information on rheumatic diseases, treatments, and available aids and support (http://www.reumacentrumtwente.nl). Additionally, the patient web portal contains a personal secure login section, where patients can find their diagnosis, current medication and medication history, blood results, actual and previous disease activity, and outcomes on quality of life related instruments. All data is accompanied by written information and (where possible) charts and graphs to show the fluctuation in scores along a timeline using colors to compare the data to norm scores. A screenshot of one of the patient web portal pages can be found in Figure 1. The purpose of this study was to assess the use, satisfaction, and the impact of the portal on the patient-provider relationship and patient empowerment, among patients suffering from rheumatoid arthritis.

## Methods

A pretest-posttest design was used, conducting a survey in the month before the web portal went online (T0), and five months after (T1).

### Patients and procedure

In total, 415 patients diagnosed with RA, conform the 1987 American College of Rheumatology classification criteria, were selected from the patient database of the Arthritis Centre Twente in Enschede, the Netherlands. Treating rheumatologists (n = 6) were asked to exclude those patients from the selection who were deceased (n = 10), or had had their last consultation longer than one year ago (n = 24). Other reasons for rheumatologists to exclude patients were: not diagnosed with RA (n = 3), severe co-morbidity (n = 4) or change of hospital (n = 1). In total, 42 patients were excluded. The 373 remaining patients were sent a personal invitation letter and a paper-and-pencil questionnaire on T0. A reminder was sent to those patients who did not respond within two weeks. One invitation was returned as undeliverable and 6 patients called or e-mailed to report that they were not interested in participation. After the pretest, all (but these 7) patients received an invitation to visit the portal and to log in with a personal account, which could be obtained in person in the clinic. Five months later, the same sample was approached with a personal letter and posttest questionnaire, with a reminder after 2 weeks, excluding the 7 patients that had withdrawn (n = 366). At T1, four questionnaires were returned as undeliverable and two patients were deceased, resulting in 360 patients that received questionnaires on both T0 and T1. This study did not need approval of the ethical review board.

### Instruments

To investigate predictors of patient web portal use, the questionnaire on T0 comprised of socio-demographics (age, gender, education level, marital status, and employment), health literacy [23,24], Internet use (access, quantity of use and self-perceived skills), and disease characteristics (moment of diagnosis, number of clinic visits in the past 6 months, self-perceived general health).

To investigate the impact of the patient web portal, five validated instruments were included on which change could be expected due to the use of the portal. *Satisfaction with care* was measured by items based on the QUOTE rheumatic patients [25]. Six items were used measuring satisfaction with the rheumatologist and the nurse practitioner, respectively. Response options ranged from “not at all” (1) to “definitely” (4). The possible range of scores for both scales was 6 to 24, with 24 representing a high satisfaction with care. Cronbach’s alphas were .83 and .87 respectively. *Trust in the rheumatologist* was measured with the Trust In Physicians short form (TRIP_sf), which is based on the Cologne-Patient-Questionnaire scale “trust in physicians” and which measures different aspects of a trusting physician-patient-interaction [26]. Items could be answered using a 5-point Likert scale ranging from “do not agree at all” (1) to “completely agree” (5), with a possible score ranging from 5 to 20, with 20 representing a high trust in the rheumatologist. Cronbach’s alpha was .93. *Self-efficacy in patient-provider communication* was assessed with the 5-item version of the Perceived Efficacy in Patient-Physician Interactions questionnaire (PEPPI-5), which assesses the subjective sense of patients’ confidence when interacting with their physicians [27]. The instrument has been translated and validated for the Dutch situation [28]. Participants respond to each question on a 5-point Likert scale ranging from “not at all confident” (1) to “very confident” (5). The range of possible scores is 5 to 25, with 25 representing the highest patient self-efficacy. Cronbach’s alpha of this instrument was .96 in our data. *Illness perception* was assessed with the Revised Illness Perception Questionnaire (IPQ-R). The subscales ‘Personal control’ (6 items), ‘Treatment control’ (5 items), and ‘Illness coherence’ (5 items) were used, which assess personal control and self-efficacy beliefs, belief in the treatment, and understanding of the illness, respectively [29]. All items can be answered on a 5-point Likert scale ranging from “strongly disagree” (1) to “strongly agree” (5), which makes the possible range of scores 6 – 30 and 5 – 25, with 30 and 25 representing the highest perceived control. Cronbach’s alphas of the subscales were .52, .72, and .76, respectively. *Medication adherence* was assessed with the Morisky Medication Adherence scale (MMA), which measures medication-taking behavior using eight items. Response categories are yes/no for seven dichotomous items and a 5-point Likert response ranging from “always” (1) to “never” (5) for the last item, which was dichotomized [30,31]. Scores were recoded, so that higher scores represent a better medication adherence, with eight representing perfect adherence. Cronbach’s alpha was .66.

The questionnaire on T1 comprised of the same measures as the pre-test, except for health literacy. To assess use and satisfaction with the patient web portal, several questions were added to the post-test, including: (1) use and moment of use of the website section and login section of the portal, (2) sharing of the personal information from the portal with others, (3) perceived ease of use, clarity, usefulness and completeness of the portal, (4) problems encountered on the portal, and what was done to solve them, (5) difficulties with understanding information in the login section, and (6) occurrence of wrong information in the login section. Additionally, questions were asked on the self-perceived impact of the portal. These questions covered the same constructs as the aforementioned instruments, but asked patients directly if they felt that the portal caused an increase, decrease, or did not change anything concerning these outcomes.

### Data analysis

Analyses were performed using the Statistical Package for the Social Sciences (IBM SPSS Statistics 20). To analyze differences in age and gender at baseline between patients who did return the questionnaire at T0 and/or T1 and those who did not, the Mann–Whitney test was applied for age and the chi-square for gender. Descriptive statistics were used to summarize socio-demographics, Internet-related and disease characteristics, portal use and satisfaction, and perceived impact of the portal. To explore relationships between patient characteristics and portal usage (non-use, website only use, and login use), the Kruskal-Wallis test was applied for continuous variables and the chi-square for discrete variables. Whenever a significant difference was found, pairwise comparisons were performed to further analyze differences between groups, using Mann–Whitney or chi-square tests. Additionally, multi-nominal logistic regression was used to analyze which variables uniquely predicted patient web portal use. To analyze the effect of portal use, Analysis of Covariance was used, in which scores on T1 of non-users, website users and login users were compared, including their T0 scores as covariate. For all analyses, p-values <0.01 (two-tailed) were used as criterion for statistical significance, as multiple comparisons were executed.

## Results

### Respondents

Of the 372 patients who received the questionnaire at T0, 259 (70%) sent it back completed. At T1, 360 patients were sent the questionnaire, of which 214 (59%) completed it. A total of 194 (54%) patients completed both questionnaires. There were no differences in age or gender between responders and non-responders on T0. At T1 and in the paired samples, there were no differences between responders and non-responders in gender, but the mean age of responders was 4.2 years higher (*P* = .01) and 3.6 years higher (*P* = .02), respectively.

### Patient web portal use

Of all respondents on T1, more than half (54%) reported to have used the portal, and 86 respondents (40%) reported to have logged in to view their personal information (Table 1). Of all respondents with Internet access, 70% had used the portal and 54% had logged in. Lack of Internet access was the most frequent reason for not using the portal (n = 56). Other reasons not to have used the portal were: “I planned to but didn’t have time yet” (n = 30), “I’m not interested” (n = 19), “I tried, but something went wrong” (n = 7), and “I don’t know how to visit the portal” (n = 5).

**Table 1:** Patient web portal usage of respondents at T1 (n = 214)

**Patient web portal use**	**All respondents (n = 214)**		**Respondents with home Internet access (n = 158)**	
	**n**	**(%)**	**n**	**(%)**
Respondents who used the web portal	115	(54)	111	(70)
1 time	41	(19)	38	(24)
2 times	47	(22)	46	(29)
3 times or more	27	(13)	27	(17)
Respondents who used the website only	29	(14)	26	(16)
Respondents who logged in	86	(40)	85	(54)

Of the respondents who logged in on the portal, 60 (70%) reported to do this in the week before a consultation with their rheumatologist or nurse practitioner. Sixteen respondents (19%) reported doing this after their consultation (data not shown in Table). Of the respondents who logged in on the portal, 29 (34%) shared their personal information with a family member.

### Predictors of patient web portal use

Table 2 shows the personal and Internet-related characteristics of the respondents at T1. The overall mean age was 62 (SD = 13.3), ranging from 20 to 86 years old. Two thirds of the respondents were female, which is representative for our population. Overall, respondents reported using the Internet regularly, but only a minority (31%) rated their own Internet skills as “good” to “very good”. Univariate analyses showed that age, marital status, education level, employment, health literacy and all Internet-related characteristics were significantly related to portal usage. Non users were more often older, single, lower educated and unemployed. Respondents with a higher level of health literacy were more inclined to log in on the portal, as well as respondents who used the Internet more often, had more years of experience, and perceived their own skills as better.

**Table 2:** Personal and Internet-related characteristics of respondents on T1 and differences between patient web portal users and non-users (n = 214)

**Characteristic**	**Total(n = 214)**	**Non-users (n = 99)**	**Website users (n = 29)**	**Login users (n = 86)**	***P***^***1***^
Age (M, (SD))	62 (13.2)	66 (14)^ac^	63 (11)^ab^	56 (11)^bc^	.000
Gender (% female)	140 (65%)	69 (70%)	14 (48%)	57 (66%)	n.s.
Marital status (% living together)	170 (80%)	68 (70%)^ac^	26 (90%)^a^	76 (88%)^c^	.000
Education level					
low	86 (40%)	54 (55%)^ac^	8 (28%)a	24 (28%)^c^	.001
medium	89 (42%)	29 (29%)	15 (52%)	45 (52%)	
high	33 (15%)	11 (11%)	6 (21%)	16 (19%)	
missing	6 (3%)	4 (4%)	-	1 (1%)	
Employment (% working)	72 (34%)	24 (24%)^c^	10 (34%)	38 (44%)^c^	.02
Health literacy (M(SD)) (n = 157)^2^	38.6 (7.2)	36.5 (7.6)^c^	37.9 (7.2)	40.9 (6.1)^c^	.001
*Internet-related*					
Amount of Internet use					
Daily/several days a week	117 (55%)	27 (27%)^c^	17 (59%)^b^	73 (85%)^cb^	.000
One day a week or less	50 (23%)	30 (30%)	9 (31%)	11 (13%)	
Missing (no home Internet access)	47 (22%)	43 (43%)	3 (10%)	1 (1%)	
Years of Internet experience					
< 5 years	44 (21%)	21 (21%)^c^	10 (34%)^b^	13 (15%)^cb^	.001
≥ 5 years	113 (53%)	26 (26%)	16 (55%)	71 (83%)	
Missing	57 (27%)	52 (53%)	3 (10%)	2 (2%)	
Self-perceived Internet skills					
Good to very good	66 (31%)	11 (11%)^c^	6 (21%)^b^	49 (57%)^cb^	.000
Average to reasonable	75 (35%)	28 (28%)	16 (55%)	31 (36%)	
Poor	22 (10%)	13 (13%)	5 (17%)	4 (5%)	
Missing	51 (24%)	47 (47%)	2 (7%)	2 (2%)	

^1^Kruskal-Wallis or Chi-square tests.

^2^Scale ranges from 14 (low level of health literacy) to 56 (high level of health literacy); data from T0.

^a^Significant difference between non-users and website users.

^b^Significant difference between website users and login users.

^c^Significant difference between non-users and login users.

Table 3 shows an overview of health-related characteristics of the respondents on T1. Most patients had been diagnosed with RA for more than a year, and visited the rheumatology clinic regularly. The majority of respondents perceived their general health as good or excellent. None of these characteristics were significantly related to portal use.

**Table 3:** Health-related characteristics of respondents at T1 and differences between portal users and non-users (n = 214)

**Characteristics**	**Total (n = 214)**	**Non-users (n = 99)**	**Website users (n = 29)**	**Login users (n = 86)**	***P***^***1***^
Time since diagnosis					
< 5 years ago	150 (70%)	67 (68%)	22 (76%)	61 (71%)	n.s.
≥ 5 years ago	60 (28%)	28 (28%)	7 (24%)	25 (29%)	
missing	4 (2%)	4 (4%)	-	-	
Number of clinic visits in the past 6 months					
0 - 1	66 (31%)	36 (36%)	8 (28%)	22 (26%)	n.s.
2	118 (55%)	53 (54%)	16 (55%)	49 (57%)	
3 or more	23 (11%)	5 (5%)	4 (14%)	14 (16%)	
Missing	7 (3%)	5 (5%)	1 (3%)	1 (1%)	
Self-perceived general health					
Good to excellent	126 (59%)	54 (55%)	20 (69%)	52 (60%)	n.s.
Reasonable to poor	86 (40%)	43 (43%)	9 (31%)	34 (40%)	
Missing	2 (1%)	2 (2%)	-	-	

^1^Chi-square tests.

Further analyses with multi-nominal logistic regression, showed that all variables together explained 59% of the variance (R^2^ = .59 (Nagelkerke), model *χ*^2^(22) = 94.04, *P* < .001). Patient web portal use was significantly predicted by age (*b* = .09, Wald *χ*^2^(1) = 4.72, *P* = .03), with younger respondents being more inclined to use the portal. Logging in at the portal was significantly predicted by self-perceived Internet skills (*b* = −.96, Wald *χ*^2^(1) = 4.74, *P* = .03) and amount of Internet use (*b* = −.70, Wald *χ*^2^(1) = 6.07, *P* = .01).

### Satisfaction with the patient web portal

The portal was positively appraised and most login users found their personal information “fairly easy” to “very easy” to understand (Table 4). When logging in to the portal, 15 respondents experienced a single problem. As a result, three respondents left the portal, three asked for help, and nine kept trying until they succeeded. Two respondents reported reoccurring problems when logging in to the portal. One of them requested a new account, and one called the web host. Nine respondents who had logged in to the portal reported finding incorrect information. In all cases, this concerned medication or blood test information that was outdated. Three respondents mentioned this during a consultation with their doctor and one participant called the hospital. Five respondents reported not taking any action (yet) because: “I thought it wasn’t important”, “I didn’t know who to contact”, and “I was too insecure to contact anyone”.

**Table 4:** Appraisal, comprehension and accuracy of the login part of the patient web portal (n = 86)

	**M (S.D.)**	**n (%)**
*Appraisal of the login part (n = 64-75)*^*1*^		
Ease of use	4.4 (.8)	
Clarity	4.3 (.7)	
Usefulness	4.3 (.7)	
Completeness	4.1 (.9)	
*Comprehension of the login pages (n = 63-72)*^*2*^		
DAS28 (disease activity)	3.4 (.7)	
Medication (history)	3.5 (.6)	
Blood results	3.5 (.5)	
Feedback on monitored data	3.5 (.6)	
*Encountered problems when logging in on the patient web portal*		
1 problem		15 (17)
2 problems		2 (2)
Found incorrect (our-of-date) information		9 (10)

^1^Answer options ranged from 1 (very negative) to 5 (very positive).

^2^Answer options ranged from 1 (very difficult) to 4 (very easy).

### Subjective impact of the patient web portal

Several positive changes were perceived by patients who had logged on to the portal (Table 5). A large part of the respondents felt that they were more involved in their treatment and that they understood their treatment better due to the patient web portal. One third of all login users felt that the quality of care was higher as a result of the portal. Also, according to a large part of the respondents, knowledge about the disease, understanding of what care providers explain, communication with the care providers, and trust in the care providers was increased. Additionally, some patients reported to search less for health information by themselves, as a result of the information provided by the hospital-based portal. Only one participant perceived a negative change, he/she felt less involved in the treatment due to the patient web portal. No further adverse effects were reported by the participants.

**Table 5:** Perceived impact of the patient web portal according to users (n = 115)

**Empowerment-related variables**	**Website users (n = 29) n (%)**	**Login users (n = 86) n (%)**
Using the patient web portal *increased* my …		
Involvement in the treatment	1 (3%)	38 (44%)
Knowledge about the treatment	2 (7%)	32 (37%)
Quality of care	2 (7%)	25 (29%)
Knowledge about the disease	2 (7%)	21 (24%)
Understanding of what care providers explain	-	21 (24%)
Self-efficacy in communication with care providers	-	16 (19%)
Trust in my care provider	-	14 (16%)
Insight into the need of medication therapy	1 (3%)	12 (14%)
Medication adherence	-	8 (9%)
Communication with others about my disease	-	6 (7%)
Number of online searches for health information	-	4 (5%)
Using the patient web portal *decreased* my …		
Number of online searches for health information	-	15 (17%)
Worries about my health	1 (3%)	3 (3%)
Involvement in the treatment	-	1 (1%)

### Pre-post test results on impact of the patient web portal

Analyses of Covariance revealed that the T1 scores on the impact outcome measures did not differ among the three groups (non-users, website only users and login users) (Table 6). Website users and login users did not show a larger improvement in the patient-provider relationship and on empowerment than non-users did, as we would have expected. It should be noted, however, that ceiling effects at T0 were found for four out of eight outcome measures. A ceiling effect is present when at least 15% of respondents scored the highest possible score on the scale. Our results showed that 45% of the respondents scored the highest possible score on satisfaction with the rheumatologist, 52% on satisfaction with the nurse, 30% on trust in the rheumatologist and 29% on self-efficacy in the patient-provider communication. This shows that room for improvement on these measures was limited in our sample. Additionally, 56% of the respondents had a score of 7 on medication adherence, with a highest possible score of 8; while this cannot be defined as a ceiling effect, room for improvement was limited on this outcome as well.

**Table 6:** Effects of the website and login part on empowerment-related outcomes (n = 214)

	**Non-users**		**Website users**		**Login users**		***P***^***1***^
	**(n = 52–81)**		**(n = 18–24)**		**(n = 68–80)**		
	**T0**	**T1**	**T0**	**T1**	**T0**	**T1**	
Satisfaction with rheumatologist^2^	22.3 (2.3)	22.6 (2.3)	21.9 (2.1)	23.0 (1.6)	22.4 (2.1)	22.6 (2.0)	n.s.
Satisfaction with nurse^2^	22.5 (2.4)	22.6 (2.4)	21.9 (2.1)	23.0 (2.0)	22.4 (2.5)	22.8 (2.0)	n.s.
Trust in the rheumatologist^3^	17.1 (2.2)	17.5 (2.3)	16.8 (2.3)	16.3 (3.2)	17.3 (2.3)	17.4 (2.3)	n.s.
Perceived self-efficacy in patient-provider communication^4^	21.3 (3.1)	21.8 (3.3)	20.7 (2.8)	20.9 (3.2)	21.2 (3.5)	21.3 (3.2)	n.s.
Illness perception							
Personal control^5^	18.8 (2.8)	18.9 (3.4)	19.0 (2.9)	19.4 (3.1)	19.3 (3.3)	19.6 (3.9)	n.s.
Illness coherence^4^	16.1 (3.1)	16.2 (3.6)	16.5 (3.6)	17.7 (3.8)	17.5 (3.7)	17.4 (3.5)	n.s.
Treatment control^4^	18.8 (2.6)	18.5 (3.0)	18.9 (3.1)	18.8 (2.0)	19.0 (2.6)	19.2 (2.5)	n.s.
Medication adherence^6^	6.5 (1.4)	6.7 (1.2)	5.5 (1.3)	6.5 (1.3)	6.3 (1.5)	6.5 (1.2)	n.s.

^1^Analyses of Covariance; ^2^possible range: 6 – 24; ^3^possible range: 5 – 20; ^4^possible range: 5 – 25; ^5^possible range: 6 – 30; ^6^possible range: 1 (severely lacking adherence) – 8 (perfect adherence).

## Discussion

Our study shows that there is a large interest among RA patients for a hospital-based rheumatology web portal. More than half of the respondents with Internet access logged in to the portal, to view their personal data. Of all the non-users in our sample, only 10% reported not wanting to use the portal because they were not interested. The other non-users either had no access to the Internet, or intended to visit the portal in the future. Reported usage from other studies on patient web portals with EMR access varies from only 6% [32] to up to 86% [33]. However, it is difficult to compare the results of our study with these previous studies, as they differ widely in types of patient groups and in the additional services that were provided. Notable is that the portion of patients in our study that logged in to view their personal information is much larger than the portion that only viewed the general information on the patient web portal. Previous studies have also found that personal information, and especially laboratory results, are more useful than general information to patients with chronic conditions [13]. Concordantly, we can conclude that our portal with EMR access foresees in a need in these patients.

One of the aims of our study was to investigate determinants of use of the application. Out of all the included variables, only age was a significant predictor of general portal use: younger patients were more inclined to visit the portal. This corresponds to what was found in much of the previous research on predictors of use of online applications [16,17,34]. Because the mean age of our sample was 62, our data shows that older generations in the Netherlands actively use the Internet, the proportion of which will only increase in the upcoming generations. As expected, self-perceived Internet skills and amount of Internet use significantly predicted logging in on the patient web portal. Previous research focusing on adoption of patient web portals, has also found that computer literacy can be a barrier in the uptake of health technologies [35,36]. It would be interesting to study whether more active encouragement and guidance from care providers towards patients with low (e)health literacy would affect their interest and use of the application.

Our portal was designed with a strong focus on the end-user, in which patients were invited to be actively involved in the determination of the content and the design of the portal. Also, together with rheumatology care providers, we made an effort to present up to date DAS28 and lab results in a clear overview. Previous studies have shown that care providers are hesitant about patient EMR access because it could confuse patients [21,37,38], but our results show that patients found the login part of the portal usable and understandable. Additionally, “mistakes” that were found in the patient-accessible EMR only concerned data that was slightly outdated and the few patients that experienced this handled these issues very well. While it should be noted that our patient-accessible EMR contained only a selection from the full medical records, it seems that when implemented carefully, expected drawbacks hardly occur. This confirms that a user-centered design is beneficial in the development of patient web portals. Further research should also explore care providers’ experiences with patients who use the portal, and the changes in work flow that they perceive, in order to determine to what extent the patient web portal changes health care processes.

Evaluating the impact of the patient web portal, we could not find significant differences over time in empowerment. Nevertheless, patients that logged in to the portal did report to perceive a larger involvement in, and understanding of, their treatment. Patients also reported that using the login part of the portal improved their knowledge of their disease and increased their capability to understand their care providers. Most patients reported using the login service prior to a consultation, which could indicate that they used it to prepare for the conversation with their doctor or nurse [34,38]. Two recent systematic reviews on the effects of patient web portals with EMR access show that only a few other studies have included empowerment-related outcomes into their evaluations, with small and inconclusive results [13,10]. Tuil et al. [9], who conducted a study among patients undergoing IVF treatment, could not detect any enhancement in patient empowerment over time either. Ross et al. [15], who evaluated a portal with EMR access among patients with congestive heart failure, found improvements in medication adherence and a small trend in increased self-efficacy and satisfaction with patient-provider communication. In our study, the lack of change over time might be explained by the timeframe between both measurements. If the posttest had been assessed at a later moment in time, through which patients could have used the portal more regularly, (especially in relation to more consultations) effects would perhaps have been more visible. Moreover, the instruments used might not have been responsive enough to measure a difference. Large ceiling effects were found on the outcomes before using the patient web portal, leaving little room for improvement.

When interpreting these results, we should take note of some limitations. First, a response bias might have occurred. It is conceivable that patients who completed the questionnaires were already satisfied and involved patients, preempting any measurable increase of the empowerment-related outcomes. Such response bias should also be mentioned in the light of the usage, satisfaction and perceived effect of the portal; it is possible that patients who were more interested in online information and support in the first place were more intended to use the portal, to complete our surveys and to value the portal positively. Second, this study relied on self-report measurements; however we are not sure if patients are able and motivated to make reliable assessments of their behavior. Future studies using less subjective measures, such as observations of doctor-patient communications or pill-counting boxes, could reveal whether patients are actually more knowledgeable about their disease, better able to speak up to their doctor and more adherent to medication. Also, observing patients’ portal usage during assignments might provide a more valuable assessment of actual web portal usability. From a previous study, it appeared that patients are not always aware of the mistakes they make when using the Internet for health-related purposes [39]. To further enhance the use and the impact of the portal, more attention might be paid to its content during consultations, so that patients might learn how to use the information from their records for their own benefit [40,41].

## Conclusions

In conclusion, a hospital-based rheumatology patient web portal with EMR access offers rheumatoid arthritis patients usable and understandable access to personal information. While the actual impact on patient empowerment is difficult to measure, a large portion of patients does feel more informed and involved in their own treatment due to the portal.

## Competing interests

The authors declare that they have no competing interests.

## Authors’ contributions

RV carried out the evaluation study, analyzed the data and drafted the manuscript. CD and ET participated in the design of the study and helped to draft the manuscript. HV, WD enabled the study, helped with data acquisition and amended the manuscript in the final stage. ML enabled the study, participated in the design and amended the manuscript in the final stage. All authors read and approved the final manuscript.

## Pre-publication history

The pre-publication history for this paper can be accessed here:

http://www.biomedcentral.com/1471-2474/15/102/prepub
